# Association between systemic omega-3 polyunsaturated fatty acid levels, and corneal nerve structure and function

**DOI:** 10.1038/s41433-022-02259-0

**Published:** 2022-09-26

**Authors:** Alexis Ceecee Britten-Jones, Jennifer P. Craig, Andrew J. Anderson, Laura E. Downie

**Affiliations:** 1 Department of Optometry and Vision Sciences, The University of Melbourne, Parkville, Australia; 2 Department of Ophthalmology, New Zealand National Eye Centre, The University of Auckland, Auckland, New Zealand

**Keywords:** Corneal diseases, Medical research

## Abstract

**Background:**

Omega-3 polyunsaturated fatty acids (PUFAs), eicosapentaenoic acid (EPA) and docosahexaenoic acid (DHA), have anti-inflammatory and neuroprotective properties. This study sought to determine the relationship between corneal parameters and systemic omega-3 fatty acid levels.

**Methods:**

Forty-seven participants with no/mild peripheral neuropathy (26 with diabetes and 21 without) underwent comprehensive ocular surface and systemic PUFA assessments. Corneal anatomical parameters were assessed using in vivo confocal microscopy. Corneal sensitivity was measured using non-contact esthesiometry. Relationships between systemic PUFA levels and corneal parameters were evaluated with multiple linear regression, adjusted for age, sex, neuropathy symptom score, and presence of diabetes and dry eye disease. The relationship between corneal nerve fibre length (CNFL) and corneal sensitivity threshold was evaluated.

**Results:**

The median Omega-3 Index, a measure of erythrocyte EPA and DHA, was 5.21% (interquartile range: 4.44–5.94%) in the study population. Mean ( ± SD) CNFL was 13.53 ± 3.37 mm/mm^2^. Multiple linear regression showed that Omega-3 Index (*β* = 0.33; *p* = 0.02), age (*β* = −0.46; *p* = 0.001) and diabetes (*β* = −0.30; *p* = 0.03) were independently associated with CNFL (*R*^*2*^ = 0.39, *p* = 0.002). In a separate model, DHA (*β* = 0.32; *p* = 0.027) and age (*β* = −0.41; *p* = 0.003) were associated with CNFL (*R*^*2*^ = 0.37, *p* = 0.003). Neither systemic EPA nor omega-6 fatty acid levels correlated with CNFL. There was no association between PUFA levels and corneal sensitivity or corneal immune cell density. A negative correlation was found between CNFL and corneal sensation thresholds to a cooled stimulus in diabetes participants, in the central (ρ = −0.50; *p* = 0.009) and peripheral (ρ = −0.50; *p* = 0.01) cornea.

**Conclusions:**

A positive relationship between the systemic Omega-3 Index and corneal nerve parameters suggests omega-3 PUFA intake may influence corneal nerve architecture.

## Introduction

Peripheral nerve damage is a potential complication of several systemic conditions, most commonly diabetes mellitus. In diabetes, loss of corneal nerves has been shown to occur prior to the onset of distal sensorimotor polyneuropathy (DSPN) [1]. Both corneal nerve structure evaluated with in vivo confocal microscopy (IVCM) and corneal sensory function quantified using esthesiometry are validated surrogate markers of peripheral neuropathy [2, 3].

The management of peripheral neuropathy typically focuses on treating the underlying condition(s). For most etiologies, including diabetes, interventions that can modulate peripheral nerve health in early-stage disease need to be identified to advance current clinical care. There is growing evidence that dietary omega-3 and omega-6 polyunsaturated fatty acids (PUFAs) play a role in modulating peripheral nerve health [4].

Omega-3 PUFAs are essential fatty acids that cannot be synthesized de novo in humans and must be derived from food or supplementation. Metabolism of long-chain omega-3 PUFAs, docosahexaenoic acid (DHA) and eicosapentaenoic acid (EPA), yields a range of lipid mediators that have anti-inflammatory and/or neuroprotective effects [4, 5]. In contrast, increasing dietary intake of long-chain omega-6 PUFAs generally biases metabolic pathways towards the breakdown of arachidonic acid (AA), and the subsequent production of pro-inflammatory mediators that can exacerbate neural dysfunction [6].

Systemic omega-3 fatty acid levels can be quantified clinically using the Omega-3 Index, which provides a measure of blood DHA and EPA levels; specifically, it represents the DHA and EPA content expressed as a percentage of the total weight of erythrocyte membrane fatty acids [7, 8]. The Omega-3 Index is a validated marker of cardiovascular health [9] and has also been associated with better insulin sensitivity and metabolic profiles in overweight men [10]. Preclinical and clinical studies have shown that oral omega-3 PUFA supplementation can attenuate corneal nerve loss and promote nerve regeneration in type 1 diabetes [11, 12] and dry eye disease [13]. Dietary modification with omega-3 PUFAs reduces ocular surface inflammation and modulates tear inflammatory cytokine levels [14, 15]. However, it is currently unclear whether a relationship exists between an individual’s basal systemic omega-3 PUFA level and corneal nerve structure and/or function, or corneal immune cell density.

The aim of this study was to evaluate the relationship between systemic fatty acid levels and corneal morphological and functional parameters. This study also examined alterations in temperature-modulated corneal structure-function relationships in individuals with and without diabetes.

## Materials and methods

This prospective, cross-sectional study was undertaken in the Department of Optometry and Vision Sciences, The University of Melbourne (Victoria, Australia) between March 2018 and January 2019. The study received institutional ethical approval (IDs #1749830 and HREC/17/SVHM/236) and was performed in accordance with the principles of the Declarations of Helsinki. All participants provided written informed consent to participate.

Eligible participants were aged 18 years or over and scored less than 16 on the Norfolk Quality of Life-Diabetic Neuropathy (QoL-DN) questionnaire [16]. A score of 16 and above identifies diabetic neuropathy with 79% sensitivity and 85% specificity [16]. Both healthy individuals and those with either type 1 or 2 diabetes were recruited, to encompass a broad spectrum of corneal nerve parameter measurements in the study population. For 14 participants with type 1 diabetes, data included the present analysis derived from the baseline data from a previously reported clinical trial [11]. Participants with diabetes provided details of their most recent HbA1c values, within the past three months.

Participant exclusion criteria were: i) history of rigid contact lens wear; ii) known hypersensitivity to ocular agents required for the study; iii) any of: active ocular infection or inflammation except mild dry eye disease, history of recurrent herpetic keratitis or active disease within six months of enrolment, corneal disorders/abnormalities that may affect corneal nerve integrity; iv) a diagnosis of peripheral neuropathy from any cause other than diabetes; v) best corrected visual acuity of worse than 6/12 in either eye; vi) women who were pregnant or breastfeeding. Soft contact lens wearers were asked to refrain from lens wear for at least 24 h before examinations. No lubricating eye drops were permitted within two hours of examination.

### Ocular surface examination

Dry eye symptoms were captured using the Ocular Surface Disease Index (OSDI) [17]. Tear osmolarity was measured bilaterally with the TearLab™ Osmolarity System (TearLab™ Corp., San Diego, CA). Ocular surface health was assessed from slit lamp examination, with clinical grading performed using the Efron scale in 0.1 increments [18]. Tear break-up time (TBUT) was measured using fluorescein (Amcon dry eye tests, DET Nomax Inc., St Louis MO, USA) and quantified as the average of three measures from each eye. Corneal fluorescein staining was evaluated immediately after TBUT measurements, followed by conjunctival staining with lissamine green (GreenGlo, HUB pharmaceuticals, CA, USA). Corneal and conjunctival staining were graded using the Oxford scale, ranging from 0 to 5 [19], in 0.1 increments [20].

### Corneal sensation

Corneal sensation was measured with a non-contact corneal esthesiometer (SDZ Esthesiometer, SDZ Electronics, Auckland, New Zealand), using an established protocol [11]. The slit lamp-mounted device has a brass stimulus jet with a 0.5 mm bore that delivers a 0.9-second air-stimulus (ranging from 0.1 to 5.0 mbar, in 0.1 mbar increments) over a corneal area of approximately 0.8 mm^2^.

Testing was performed in a temperature- and humidity-controlled room (temperature: 20 ± 3 °C; humidity: 50 ± 5%). The right eye of all participants was assessed, except in two cases of prior unilateral ocular injury, where left eyes were examined. Sensation thresholds were measured for the central cornea (at the apex) and the peripheral cornea (1 mm above the inferior limbus), for both room-temperature (23–24 °C) and cooled (18–19 °C) air stimuli.

Two randomly interleaved staircases (1-up, 1-down, step size 0.1 mbar) (MATLAB, version 9.2 (R2017a), The MathWorks Inc. Natick, MA, USA) were employed for assessing sensation thresholds. Participants verbally indicated either ‘felt’ or ‘not felt’ to indicate their detection or otherwise of the stimulus. The final threshold was calculated as the average of all staircase reversals, excluding the first reversal in each staircase, once each staircase had reached at least three reversals. If participants responded ‘felt’ to two stimuli at 0.1 mbar, false positive checks (i.e., no stimulus delivered) were undertaken to ensure participants’ reliability [11]. If passed, participants were considered to have a threshold of <0.1 mbar if either one or two subsequent “felt” responses were made, or a threshold of 0.1 mbar if two “not felt” responses were made, to subsequently presented 0.1 mbar stimuli.

### Corneal in vivo confocal microscopy (IVCM)

Corneal IVCM was performed on the same eye with the Heidelberg Retinal Tomograph III with Rostock Corneal Module (Heidelberg Engineering GMB, Dossenheim, Germany), using an established protocol [11]. This involved participants progressively re-fixating over 12 prescribed locations, arranged in a 3-down and 4-across matrix situated 135 cm from the eye. Multiple sequence scans were used to capture images in the central cornea (at the corneal apex) and peripheral cornea (1–1.5 mm above the inferior limbus). Between 300 and 600 images (each 400 × 400 μm), across an area of approximately 2 mm^2^, were captured in each corneal region for each participant.

### Image analyses

For each participant, corneal sub-basal nerve plexus (SBNP) parameters were quantified from 12 randomly-selected images of the central cornea and eight images of the peripheral cornea [21]. Images that were blurred or showed vignetting effects were removed from the potential analysis sample prior to randomization. Images were checked to ensure less than 20% overlap in the imaging region.

Central corneal SBNP parameters were quantified using ACCMetrics (v2, University of Manchester; UK) [22] for corneal nerve fibre length (CNFL, mm/mm^2^), corneal nerve fibre density (CNFD, nerves/mm^2^), corneal nerve branch density (CNBD, branches on main fibre/mm^2^), and corneal nerve total branch density (CTBD; total branches/mm^2^). Peripheral CNFL was manually quantified using the NeuronJ plugin on ImageJ (US National Institutes of Health, USA) [23, 24]. Peripheral CNFL was calculated as the sum of total nerve length divided by 0.16 mm^2^.

Corneal immune cells were analyzed from the same IVCM images. Cell counts were performed using an ImageJ plugin [24]. Immune cells were identified by their highly reflective bodies, with or without dendrites, in the corneal SBNP and classified into three morphological phenotypes [25], as described by Lagali et al. [26].

### Systemic fatty acid levels

Systemic fatty acid profiles were analyzed using dried blood spot (DBS) tests (Waite Lipid Analysis Service (WLAS), University of Adelaide, South Australia, Australia) [27, 28]. Approximately 3 mL of capillary blood was collected and spotted onto the proprietary PUFAcoat™ test cards, according to the manufacturer’s instructions. The cards were air dried, placed in sealed cellophane bags and stored with desiccants in a dark, temperature-controlled chamber (21–22 °C). All DBS testing kits were sent for laboratory analysis within four weeks of collection. The analyzed fatty acid parameters were: 20:5n-3 (EPA), 22:6n-3 (DHA), 20:4n-6 (AA), and total omega-3 and omega-6 PUFA levels. The Omega-3 Index (%) was defined as the total percentage of EPA and DHA present in erythrocyte phospholipid membranes [27]. Fatty acid intake was also validated using a dietary questionnaire, the Clinical Omega-3 Dietary Survey; these results are reported elsewhere [28].

### Statistical analysis

In the absence of existing literature considering a relationship between systemic omega-3 fatty acid levels and corneal nerve parameters, sample size calculation was based on published data on the corneal structure-function relationship. For an estimated correlation coefficient of 0.40 [29], with 80% power at a confidence level of 95%, 47 participants were required in the study.

Statistical analyses were performed using R Foundation for Statistical Computing (version 4.0.0. Vienna, Austria: R Core Team)[30] and SPSS Statistics (Version 23.0. Armonk, NY: IBM Corp.). For corneal sensitivity measurements, sub-threshold values (<0.1 mbar) were replaced by the lower limit divided by two (i.e., 0.05 mbar) [31]. The presence of dry eye disease was classified dichotomously based on the TFOS DEWS II diagnostic criteria using data from both eyes as: OSDI score ≥13, and one of: i) tear osmolarity ≥308 mOsm/L in either eye or an interocular difference >8 mOsm/L; ii) TBUT < 10 s; or iii) clinical signs of >5 corneal spots with sodium fluorescein or >9 conjunctival spots with lissamine green (interpreted as grade 1.5 or higher using the Oxford grading in the present study) [32].

Data normality was assessed using the Shapiro-Wilk test. Descriptive statistics are summarized as mean ± SD for normally distributed data, or median (inter-quartile range, IQR) for non-normal data. Inter-group comparisons were analyzed using the independent sample *t*-test for normally distributed variables, the Mann-Whitney-U test for non-normally distributed variables, and the Fisher’s exact test (two-tailed) for categorical variables. Homogeneity of variance between groups was evaluated using the F-test.

The association between CNFL and corneal sensation thresholds was evaluated using Spearman’s correlation coefficient (*ρ*) for non-normally distributed data. Outliers were evaluated using the ROUT method [33], and the robustness of the results was evaluated in a sensitivity analysis excluding any outliers. For all comparisons, an alpha value of 0.05 was adopted to define statistical significance.

The relationship between systemic PUFA levels and corneal nerve and immune cell parameters was evaluated using multiple linear regression. Fixed predictor variables included fatty acid parameters, age, sex, Norfolk QoL-DN score, presence of diabetes, and presence of dry eye disease. For the diabetes subgroup, univariate models examined whether diabetes duration, diabetes type, and HbA1c were factors influencing the dependent variables to determine model selection. For non-normally distributed dependent variables, a log-transformation was performed prior to model fitting. For corneal sensation threshold as the dependent variable, tear osmolarity was incorporated as an additional predictor [34].

## Results

### Participant characteristics

Table 1 summarizes the participant demographics, ocular surface parameters, and systemic PUFA profiles. The median age of study participants was 48 years (IQR: 30–64 years), and 63% of participants were female. Most participants (87%) did not have dry eye disease. The median Omega-3 Index in the study population was 5.21% (IQR: 4.44–5.94%), and the median systemic omega-6 to omega-3 ratio was 5.59 (IQR: 4.50–7.32). Participants with diabetes were of similar age and sex, and had similar systemic PUFA profiles, to those without diabetes (all *p* > 0.05). Participants with diabetes had higher neuropathy symptom scores than participants without (*p* = 0.032); however, this difference in score of 2 (out of 156) was not clinically significant [16].

**Table 1:** Participant demographic and clinical characteristics.

	Control	Diabetes	Overall
	(*n* = 21)	(*n* = 26)	(*n* = 47)
**Demographics**			
Age, years	48 (26–64)	49.0 (30–63)	48 (30–64)
Sex, number of females, *n* (%)	14 (67%)	15 (58%)	29 (63%)
Type 1 diabetes, *n* (%)	-	16 (62%)	-
HbA1c, %	-	7.0 (6.6–8.0)	-
Diabetes duration, years	-	13 (6–22)	-
Norfolk QoL-DN score, /156	1.0 (0.0–2.0)	4.5 (1.0–7.8)	2.0 (0–6.0)
**Ocular surface parameters**			
Diagnosis of DED, *n* (%)	2 (9.5)	4 (15.4)	6 (13%)
OSDI score, /100	6.7 ± 7.1	7.4 ± 8.0	7.1 ± 7.5
Tear osmolarity, mOsmol/L*	299.7 ± 6.9	301.9 ± 10.8	300.9 ± 9.3
Anterior blepharitis, /4.0^†^	0.50 (0.30–1.50)	0.88 (0.45–1.71)	0.75 (0.32–1.65)
MGD, /4.0^†^	0.70 (0.40–1.45)	0.80 (0.53–1.51)	0.75 (0.48–1.52)
Conjunctival redness, /4.0^†^	0.83 (0.70–1.03)	0.80 (0.59–1.05)	0.80 (0.61–1.05)
Limbal redness, /4.0^†^	0.35 (0.25–0.65)	0.43 (0.25–0.75)	0.40 (0.25–0.73)
TBUT, seconds	10.40 (8.94–11.66)	7.40 (5.80–8.97)	8.81 (6.81–10.76)
Corneal staining, /5.0^‡^	0.15 (0.10–0.40)	0 (0–0.19)	0.10 (0.00–0.30)
Conjunctival staining, /5.0^‡^	0.15 (0.03–0.30)	0.05 (0–0.28)	0.12 (0.00–0.29)
**Systemic fatty acid profiles**			
Total omega-6, %	28.55 ± 5.44	22.58 ± 5.04	25.25 ± 5.97
Total AA (22:4n-6), %	6.77 ± 1.50	5.61 ± 1.63	6.13 ± 1.66
Total omega-3, %	4.90 (3.91–5.07)	4.21 (3.49–4.97)	4.39 (3.60–5.05)
Total EPA (20:5n-3), %	0.60 (0.45–0.73)	0.62 (0.51–0.82)	0.61 (0.47–0.80)
Total DHA (22:6n-3), %	2.05 (1.88–2.60)	1.96 (1.49–2.31)	2.00 (1.74–2.59)
Omega-3 Index, %	5.54 (4.54–6.06)	5.00 (4.25–5.68)	5.21 (4.44–5.94)
Omega-6:omega-3 ratio	6.69 (5.09–7.67)	5.27 (4.49–6.36)	5.59 (4.50–7.32)

Data are in mean ± SD or median (IQR) as appropriate, unless otherwise indicated. *Shown as the highest value of the two eyes. ^†^Using the Efron Grading Scale. ^‡^Using the Oxford grading scale. *AA* Arachidonic acid, *EPA* Eicosapentaenoic acid, *DED* Dry eye disease, *DHA* Docosahexaenoic acid, *HbA1c* Glycosylated haemoglobin, *MGD* Meibomian gland dysfunction, *Norfolk QoL-DN* Norfolk Quality of Life–Diabetic Neuropathy questionnaire, *OSDI* Ocular Surface Disease Index questionnaire, *TBUT* Tear break-up time.

### Corneal parameters and systemic omega-3 fatty acid levels

Tables 2 and 3 summarize the relationship between systemic PUFA profiles and corneal nerve and immune cell parameters, accounting for age, sex, presence of diabetes, presence of dry eye disease, and Norfolk QoL-DN score.

**Table 2:** Multiple linear regression models for variables predicting central corneal nerve fibre length (CNFL).

Variable	B (95% CI)	*β*	*p*-value
**Model 1: Omega-3 Index**			
Omega-3 index, %	0.83 (0.16 to 1.50)	0.33	**0.017**
Age, years	−0.08 (−0.13 to −0.04)	−0.46	**0.001**
Diabetes, present	−1.99 (−3.78 to −0.20)	−0.30	**0.030**
*R* = 0.601. *R*^*2*^ = 0.361. Adjusted *R*^*2*^ = 0.316. *F* = 8.10, *p* ≤ 0.0001			
**Model 2: Systemic EPA levels**			
EPA levels, %	1.31 (−0.82 to 2.70)	0.25	0.064
Age, years	−0.09 (−0.14 to −0.04)	−0.48	**0.001**
Diabetes, present	−2.20 (−4.04 to −0.36)	−0.33	**0.020**
*R* = 0.59. *R*^*2*^ = 0.35. *F* = 3.60. *p* = 0.006.			
**Model 3: Systemic DHA levels**			
DHA levels, %	1.97 (0.24 to 3.71)	0.32	**0.027**
Age, years	−0.07 (−0.12 to −0.03)	−0.41	**0.003**
Diabetes, present	−1.82 (−3.64 to 0.006)	−0.27	0.051
*R* = 0.61. *R*^*2*^ = 0.37. *F* = 3.99. *p* = 0.003.			
**Model 4: Total omega-6 fatty acid levels**			
Total omega-6 levels, %	−0.02 (−0.20 to 0.17)	−0.03	0.855
Age, years	−0.08 (−0.13 to −0.03)	−0.44	**0.004**
Diabetes, present	−2.22 (−4.39 to −0.05)	−0.33	**0.045**
*R* = 0.54. *R*^*2*^ = 0.30. *F* = 2.75. *p* = 0.025.			

Additional variables in the model not found to be related to CNFL are sex, presence of dry eye diseases, and Norfolk Quality of Life-Diabetic Neuropathy questionnaire score. **B**, unstandardised regression coefficient. **β**, standardised regression coefficient. *CI* Confidence interval, *DHA* Docosahexaenoic acid, *EPA* Eicosapentaenoic acid, *Norfolk QoL-DN* Norfolk Quality of Life-Diabetic Neuropathy questionnaire.

Statistically significant *p*-values are in bold.

**Table 3:** Multiple linear regression models for variables predicting central corneal nerve fibre density (CNFD).

Variable	B (95% CI)	*β*	*p*-value
**Model 1: Systemic Omega-3 Index**			
Omega-3 index, %	1.71 (0.37 to 3.04)	0.35	**0.014**
Age, years	−0.16 (−0.25 to −0.065)	−0.44	**0.001**
Diabetes, present	−3.51 (−7.08 to 0.06)	−0.27	0.054
*R* = 0.60. *R*^*2*^ = 0.36. *F* = 3.79. *p* = 0.004.			
**Model 2: Systemic EPA levels**			
EPA levels, %	2.42 (−0.40 to 5.23)	0.24	0.090
Age, years	−0.17 (−0.27 to −0.067)	−0.47	**0.002**
Diabetes, present	−3.92 (−7.64 to −0.21)	−0.30	**0.039**
*R* = 0.56. *R*^*2*^ = 0.31. *F* = 2.97. *p* = 0.017.			
**Model 3: Systemic DHA levels**			
DHA levels, %	4.42 (1.00 to 7.85)	0.37	**0.013**
Age, years	−0.14 (−0.23 to −0.044)	−0.39	**0.005**
Diabetes, present	−3.10 (−6.69 to 0.50)	−0.24	0.090
*R* = 0.60. *R*^*2*^ = 0.36. *F* = 3.82. *p* = 0.004.			
**Model 4: Total omega-6 fatty acid levels**			
Total omega-6 levels, %	−0.18 (−0.55 to 0.19)	−0.16	0.334
Age, years	−0.16 (−0.26 to −0.06)	−0.45	**0.003**
Diabetes, present	−4.76 (−9.06 to −0.46)	−0.36	**0.031**
*R* = 0.52. *R*^*2*^ = 0.27. *F* = 2.51. *p* = 0.037.			

Additional variables in the model not found to be related to CNFL are sex, presence of dry eye diseases, and Norfolk Quality of Life-Diabetic Neuropathy questionnaire score. **B**, unstandardised regression coefficient. **β**, standardised regression coefficient. *CI* Confidence interval, *DHA* Docosahexaenoic acid, *EPA* Eicosapentaenoic acid, *Norfolk QoL-DN* Norfolk Quality of Life-Diabetic Neuropathy questionnaire.

Statistically significant *p*-values are in bold.

The Omega-3 Index (*β* = 0.33; *p* = 0.02), age (*β* = −0.46; *p* = 0.001), and diabetes status (*β* = −0.30; *p* = 0.03) were factors associated with CNFL (Table 2). The relationship between CNFL and systemic levels of each of DHA, EPA and total omega-6 PUFAs were explored in separate models. DHA level was positively associated (*β* = 0.32; *p* = 0.027), and participant age (*β* = −0.41; *p* = 0.003) was negatively associated, with CNFL (*R*^*2*^ = 0.37, *p* = 0.003). However, neither EPA nor omega-6 PUFA levels were related to CNFL.

The Omega-3 Index, age, and diabetes status were also significantly associated with CNFD (Table 3; *R*^*2*^ = 0.36; *p* = 0.004). Systemic DHA level (*R*^*2*^ = 0.37 *p* = 0.003) was a significant factor for predicting CNFD, independent of age and diabetes status. Neither EPA nor total omega-6 PUFA levels were associated with CNFD (Table 3).

For corneal sensation, participant age and diabetes status were the only factors associated with thresholds to both room-temperature and cooled stimuli (Supplementary Tables A1, 2). Neither systemic omega-3 nor omega-6 PUFA levels were related to corneal sensation thresholds with stimuli of either temperature. None of the predictor variables were found to be significantly associated with total central corneal immune cell density (Supplementary Table A3).

**Fig. 1:**
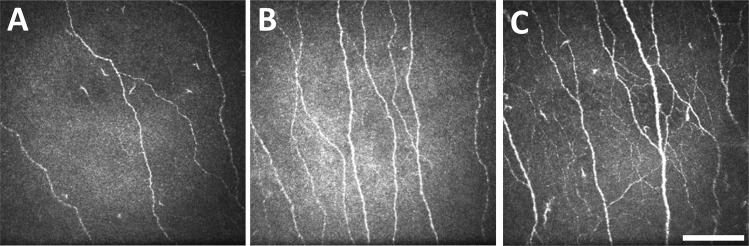
Representative in vivo confocal microscopy images of the central corneal sub-basal nerve plexus. Images shown are of three participants without diabetes, with Omega-3 Index scores of (**A**) 3.9%, (**B**) 6.0%, and (**C**) 8.4%. Scale bar=100 µm for all images.

Representative IVCM images of participants with different Omega-3 Index scores are shown in Fig. 1.

### Corneal nerve and immune cell parameters

Table 4 shows summary data for corneal nerve structural and functional parameters, and central corneal immune cell density. Measures of central corneal nerve parameters were lower for CNFL, CNBD, and CTBD in people with diabetes compared to those without (*p* < 0.05 for all comparisons). There was no inter-group difference for central CNFD or peripheral CNFL.

**Table 4:** Corneal sub-basal nerve and corneal immune cell parameters and corneal nerve function in participants.

	Control	Diabetes	Overall	*p*-value*
	(*n* = 21)	(*n* = 26)	(*n* = 47)	
**Corneal nerve structure**				
Central CNFL (mm/mm^2^)	14.71 ± 2.99	12.60 ± 3.41	13.53 ± 3.37	**0.030**
Central CNFD (nerves/mm^2^)	24.67 ± 6.05	20.99 ± 6.67	22.63 ± 6.60	0.054
Central CNBD (branches/mm^2^)	35.00 ± 12.57	23.60 ± 11.45	28.77 ± 13.11	**0.002**
Central CTBD (total branches/mm^2^)	50.88 ± 18.72	35.48 ± 16.49	42.45 ± 18.94	**0.005**
Peripheral CNFL (mm/mm^2^)	5.00 ± 2.84	4.42 ± 2.39	4.68 ± 2.59	0.460
**Corneal immune cells (ICs)**				
**Central**				
Total (cells/mm^2^)	20 (14–34)	33 (11–66)	25 (11–61)	0.600
Mature ICs (cells/mm^2^)	10 (4–18)	11 (4–26)	10 (4–25)	0.764
Immature ICs (cells/mm^2^)	12 (5–22)	12 (4–42)	12 (4–35)	0.831
Globular cells (cells/mm^2^)	0 (0–1)	0 (0–1)	0 (0–1)	0.654
**Peripheral**				
Total (cells/mm^2^)	85 (43–118)	93 (39–142)	85 (40–135)	0.661
Mature ICs (cells/mm^2^)	52 (38–96)	46 (24–78)	52 (28–93)	0.773
Immature ICs (cells/mm^2^)	18 (5–23)	16 (7–42)	17 (6–58)	0.374
Globular cells (cells/mm^2^)	0 (0–1)	0 (0–1)	0 (0–1)	0.840
**Corneal sensation thresholds**				
**Central**				
Room-temperature stimuli (mbar)	0.28 (0.18–0.35)	0.59 (0.42–0.75)	0.40 (0.25–0.64)	**<0.001**
Cooled stimuli (mbar)	0.15 (0.05–0.23)	0.48 (0.35–0.69)	0.30 (0.12–0.51)	**<0.001**
**Peripheral**				
Room-temperature stimuli (mbar)	0.33 (0.25–0.40)	0.56 (0.43–0.80)	0.45 (0.28–0.58)	**<0.001**
Cooled stimuli (mbar)	0.25 (0.05–0.30)	0.46 (0.36–0.54)	0.35 (0.25–0.50)	**<0.001**

Data are expressed as mean ± SD or median (IQR) as appropriate, unless otherwise indicated. *p-values indicate differences between the control and diabetes subpopulations. *CNBD* Corneal nerve branch density, *CNFD* Corneal nerve fibre density, *CNFL* Corneal nerve fibre length, *CTBD* Corneal nerve total branch density, *IC* Immune cell.

Statistically significant *p*-values are in bold.

Central corneal sensation thresholds were significantly higher in those with diabetes compared to those without diabetes, for both room-temperature and cooled stimuli. The same difference was observed in the peripheral cornea to stimuli of both temperatures (Table 4).

Median corneal immune cell density was 25 (IQR: 11–61) cells/mm^2^ in the central cornea and 85 (IQR: 40–135) cells/mm^2^ in the peripheral cornea. There were no significant differences in total cell density, or the density of each morphological subtype, between study populations (Table 4).

### Corneal structure function relationship

A secondary analysis was performed to consider the relationship between corneal nerve structure and function to both room-temperature and cooled stimuli, in the central and peripheral cornea. Across the study population, there was an inverse relationship between central CNFL and thresholds to both room-temperature (*ρ* = −0.41, 95% CI: −0.63 to −0.13) and cooled (*ρ* = −0.51, 95% CI: −0.72 to −0.26) air stimuli. In the peripheral cornea, CNFL was inversely correlated with thresholds to cooled air stimuli (*ρ* = −0.43, 95 % CI: −0.64 to −0.15) but not room-temperature air stimuli (*ρ* = −0.26, 95% CI: −0.51 to 0.04).

A sub-analysis in the diabetes and non-diabetes sub-populations found a negative correlation between CNFL and sensation thresholds to cooled stimuli in the diabetes group, in both the central (*ρ* = −0.50, 95% CI: −0.75 to −0.12, *p* = 0.009) and peripheral (*ρ* = −0.50, 95% CI: −0.75 to −0.11, *p* = 0.01) corneal regions (Supplementary Fig. 1). This relationship was not evident in the control group (central: *ρ* = −0.32, 95% CI: −0.66 to 0.15, *p* = 0.16; peripheral: *ρ* = −0.37, 95% CI: −0.70 to 0.09, *p* = 0.10). In both participant groups, CNFL did not correlate with sensation thresholds to room temperature stimuli in either the central or peripheral cornea (*p* > 0.05).

## Discussion

This study reports a novel positive relationship between both the systemic Omega-3 Index and erythrocyte DHA levels, with each of the corneal nerve structural parameters CNFL and CNFD, independent of age or diabetes status. In addition, this study finds a negative correlation between CNFL and corneal sensation thresholds to a cooled stimulus in people with diabetes, in both the central and peripheral cornea.

The association between systemic DHA, but not EPA, levels with corneal nerve architecture could relate to the role that DHA plays in modulating neural membrane properties [35], and the neurotrophic effects of the DHA-derived docosanoid mediator, neuroprotectin D1 (NPD1) [36]. Preclinical studies have shown that topical treatment with both DHA and NPD1 can promote corneal nerve regeneration after trigeminal nerve injury [37]. Both oral long-chain omega-3 PUFA and DHA supplements have also been reported to reduce neuropathic pain behavior in rodents [38, 39]. The present findings raise a promising notion that increasing systemic omega-3 PUFA, particularly DHA, levels could be beneficial for corneal nerves. These findings are also broadly consistent with results from clinical trials that have found that increasing omega-3 PUFA intake via oral supplementation, over several months, promotes corneal nerve regeneration [11, 12].

Our participants had an average Omega-3 Index of 5.2% (IQR: 4.4–5.9%), which is consistent with data from a systematic review showing Australians tend to have Omega-3 Indices between 4% and 6% [40], typical of a Western diet. A target Omega-3 Index range of 8–11% is considered to be beneficial during pregnancy and for cardiovascular health [8, 41]. A shift from an Omega-3 Index of 4% to 8% is estimated to reduce the risk of fatal heart disease by 30% [8]. The lower-than-ideal Omega-3 Index scores observed in the current study population are unsurprising, given that 80% of Australians do not meet the recommended omega-3 PUFA dietary intake of approximately 500 mg/day [42]. The generally low Omega-3 Indices across our participant cohort limited the ability to potentially determine a ‘target’ value for normative corneal structural parameters, stratified by age. Evaluation of a larger cohort with a wider range of Omega-3 Index scores (particularly including those under 4% and over 8%) might better inform whether the 8% cut-off used for cardiovascular markers is also an optimal threshold for corneal nerve health. Furthermore, the Omega-3 Index reliably reflects dietary DHA and EPA intake over the preceding three months [43]. We have previously validated the quantification of fatty acid intake using a dietary questionnaire, the Clinical Omega-3 Dietary Survey, and the results of this validation study have been previously published [28]. Dietary tools may be of value for longitudinally assessing systemic fatty acid intake, which can change with variations in the consumption of foods rich in omega-3 and omega-6 PUFAs. It is currently unclear whether changes to CNFL occur dynamically with alterations to systemic omega-3 PUFA levels. Supporting the potential for temporal variations, significant increases in corneal nerve parameters have been demonstrated in omega-3 PUFA intervention studies over only a few months [11, 12]. In addition, Lewis et al. found that the change from baseline in CNFL was positively associated with baseline omega-3 PUFA levels in 40 individuals with type 1 diabetes treated with oral omega-3 supplementation for 12 months [44]. Studies examining longitudinal changes in corneal nerve architecture in individuals with varying omega-3 food intake and/or omega-3 PUFA supplementation would yield further insight into the association between omega-3 intake and changes in CNFL over time.

Corneal nerves have an essential role in regulating ocular surface homeostasis and tear production. Three main functional subsets of corneal sensory nerves, comprising polymodal (60–70%), mechanical (20–30%), and cold thermoreceptor subtypes (10–15%), are recognized [45]. Conflicting findings exist in relation to the human corneal nerve structure-function relationship [46, 47]. Although reductions in corneal SBNP parameters occur prior to clinically-detectable symptoms and signs of diabetic neuropathy [1, 48], an associated loss of corneal sensation has only consistently been reported in individuals with established neuropathy [2, 49, 50]. The clinical device used to evaluate corneal nerve architecture, IVCM, enables visualization only of the corneal SBNP and not the superficial nerve terminals, whereas corneal sensitivity is influenced by nociceptors across multiple corneal layers [51].

In vitro investigations have shown that corneal cold thermoreceptors expressing TRPM8 receptors are disproportionately affected over polymodal and mechano-nociceptors in early diabetes [52]. However, whether temperature-modulated changes in corneal nerve function in humans with diabetes exist is not yet established. The present study found that, relative to healthy individuals, the corneal structure-function relationship was altered in those with diabetes, who had reduced corneal nerve structural parameters and sensitivity. These observations extend previous findings where altered corneal nerve structure has been shown in individuals with diabetes without diabetic neuropathy [2], and potentially indicate disease-related effects particularly alter corneal cold thermoreceptor function. A putative mechanism could relate to differential effects of TRPM8 containing axons that have been shown to be preferentially affected in early experimental diabetes in pre-clinical studies [52]. A decrease in CNFD in diabetes has also been associated with an increase in cold detection thresholds in the skin [53]. These findings support the hypothesis that losses of small nerve fibres may be associated specifically with changes in sensitivity to cooled temperatures.

Potential considerations relating to the interpretation of the current findings are that the participant group studied intentionally included people with and without diabetes, in order to capture a greater spectrum of corneal nerve health; this was based on a presumption that the relationship between systemic omega-3 PUFA levels and corneal nerve parameters are similar in these two subpopulations, and was confirmed in the multiple linear regression model. The peripheral neuropathy classification was based on a validated symptom questionnaire rather than neurophysiological measurements. Future investigations into the association between the Omega-3 Index and peripheral nerve function in the limbs, such as using nerve conduction studies and quantitative sensory tests, would further understanding of the relationship between systemic omega-3 PUFAs levels and peripheral nerve function.

In conclusion, this study found a positive association between the systemic Omega-3 Index and quantitative measures of corneal nerve architecture; this relationship was independent of age or diabetes status. These findings support a relationship between systemic omega-3 PUFA levels, in particular DHA, and anatomical parameters associated with corneal nerve health. Furthermore, an altered corneal structure-function relationship to cold stimuli in diabetes may indicate disease-related effects that are specific to thermosensitive sub­population(s) of corneal nerves.

## Summary

### What was known before

- Oral long-chain omega-3 fatty acid supplementation has been shown to promote corneal nerve regeneration in people with type 1 diabetes and dry eye disease. Animal studies have also shown that the long-chain omega-3 fatty acid, docosahexaenoic acid (DHA), has neuroprotective effects.

### What this study adds

- This clinical study finds that both systemic total omega-3 fatty acid levels and docosahexaenoic acid (DHA) levels are independently associated with corneal nerve anatomical parameters, independent of a person’s age or diabetes status. These new findings suggest that an individual’s omega-3 fatty acid, in particular DHA, dietary intake may influence the health of their corneal nerves.

## Supplementary information

Supplemental Tables and Figure

## Data Availability

The datasets analysed during the current study are available from the corresponding author on reasonable request, subject to approval of an ethics application amendment to permit data sharing by the relevant human research ethics committee.

## References

[1] Ziegler D, Papanas N, Zhivov A, Allgeier S, Winter K, Ziegler I (2014). Early detection of nerve fiber loss by corneal confocal microscopy and skin biopsy in recently diagnosed type 2 diabetes. Diabetes.

[2] Pritchard N, Edwards K, Dehghani C, Fadavi H, Jeziorska M, Marshall A (2014). Longitudinal assessment of neuropathy in type 1 diabetes using novel ophthalmic markers (LANDMark): Study design and baseline characteristics. Diabetes Res Clin Pr.

[3] Tavakoli M, Boulton AJ, Efron N, Malik RA (2011). Increased Langerhan cell density and corneal nerve damage in diabetic patients: role of immune mechanisms in human diabetic neuropathy. Cont Lens Anterior Eye.

[4] Zhang AC, De Silva MEH, MacIsaac RJ, Roberts L, Kamel J, Craig JP (2020). Omega-3 polyunsaturated fatty acid oral supplements for improving peripheral nerve health: a systematic review and meta-analysis. Nutr Rev.

[5] Calder PC, Grimble RF (2002). Polyunsaturated fatty acids, inflammation and immunity. Eur J Clin Nutr.

[6] Boyd JT, LoCoco PM, Furr AR, Bendele MR, Tram M, Li Q (2021). Elevated dietary ω-6 polyunsaturated fatty acids induce reversible peripheral nerve dysfunction that exacerbates comorbid pain conditions. Nat Metab.

[7] Block RC, Harris WS, Pottala JV (2008). Determinants of Blood Cell Omega-3 Fatty Acid Content. Open Biomark J.

[8] Harris WS, Del Gobbo L, Tintle NL (2017). The Omega-3 Index and relative risk for coronary heart disease mortality: Estimation from 10 cohort studies. Atherosclerosis.

[9] Harris WS, Von Schacky C (2004). The Omega-3 Index: A new risk factor for death from coronary heart disease?. Prev Med.

[10] Albert BB, Derraik JGB, Brennan CM, Biggs JB, Smith GC, Garg ML (2014). Higher omega-3 index is associated with increased insulin sensitivity and more favourable metabolic profile in middle-aged overweight men. Sci Rep.

[11] Britten-Jones AC, Kamel JT, Roberts LJ, Braat S, Craig JP, MacIsaac RJ (2021). Investigating the neuroPRotective effect of Oral Omega-3 Fatty acid Supplementation in type 1 diabetes (nPROOFS1): a randomised, placebo-controlled trial. Diabetes.

[12] Lewis EJH, Perkins BA, Lovblom LE, Bazinet RP, Wolever TMS, Bril V (2017). Effect of omega-3 supplementation on neuropathy in type 1 diabetes: A 12-month pilot trial. Neurology.

[13] Chinnery HR, Naranjo Golborne C, Downie LE (2017). Omega-3 supplementation is neuroprotective to corneal nerves in dry eye disease: a pilot study. Ophthalmic Physiol Opt.

[14] Brignole-Baudouin F, Baudouin C, Aragona P, Rolando M, Labetoulle M, Pisella PJ (2011). A multicentre, double-masked, randomized, controlled trial assessing the effect of oral supplementation of omega-3 and omega-6 fatty acids on a conjunctival inflammatory marker in dry eye patients. Acta Ophthalmol.

[15] Deinema LA, Vingrys AJ, Wong CY, Jackson DC, Chinnery HR, Downie LE (2017). A Randomized, Double-Masked, Placebo-Controlled Clinical Trial of Two Forms of Omega-3 Supplements for Treating Dry Eye Disease. Ophthalmology.

[16] Vinik EJ, Hayes RP, Oglesby A, Bastyr E, Barlow P, Ford-Molvik SL (2005). The development and validation of the Norfolk QOL-DN, a new measure of patients’ perception of the effects of diabetes and diabetic neuropathy. Diabetes Technol Ther.

[17] Schiffman RM, Christianson MD, Jacobsen G, Hirsch JD, Reis BL (2000). Reliability and validity of the Ocular Surface Disease Index. Arch Ophthalmol.

[18] Efron N. Appendix A - Grading scales for contact lens complications. In: Efron N (ed). Contact Lens Complications (Third Edition). W.B. Saunders: London; 2012. pp 301–5.

[19] Bron AJ, Evans VE, Smith JA (2003). Grading of corneal and conjunctival staining in the context of other dry eye tests. Cornea.

[20] Bailey IL, Bullimore MA, Raasch TW, Taylor HR (1991). Clinical grading and the effects of scaling. Invest Ophthalmol Vis Sci.

[21] Vagenas D, Pritchard N, Edwards K, Shahidi AM, Sampson GP, Russell AW (2012). Optimal image sample size for corneal nerve morphometry. Optom Vis Sci.

[22] Dabbah MA, Graham J, Petropoulos IN, Tavakoli M, Malik RA (2011). Automatic analysis of diabetic peripheral neuropathy using multi-scale quantitative morphology of nerve fibres in corneal confocal microscopy imaging. Med Image Anal.

[23] Meijering E, Jacob M, Sarria JC, Steiner P, Hirling H, Unser M (2004). Design and validation of a tool for neurite tracing and analysis in fluorescence microscopy images. Cytom A.

[24] Schindelin J, Arganda-Carreras I, Frise E, Kaynig V, Longair M, Pietzsch T (2012). Fiji: an open-source platform for biological-image analysis. Nat Methods.

[25] Britten-Jones AC, Rajan R, Craig JP, Downie LE. Quantifying corneal immune cells from human in vivo confocal microscopy images: Can manual quantification be improved with observer training? Exp Eye Res. 2022;216:108950. 10.1016/j.exer.2022.108950 35065982

[26] Lagali NS, Badian RA, Liu X, Feldreich TR, Ärnlöv J, Utheim TP (2018). Dendritic cell maturation in the corneal epithelium with onset of type 2 diabetes is associated with tumor necrosis factor receptor superfamily member 9. Sci Rep.

[27] Liu G, Muhlhausler BS, Gibson RA (2014). A method for long term stabilisation of long chain polyunsaturated fatty acids in dried blood spots and its clinical application. Prostaglandins Leukot Ess Fat Acids.

[28] Zhang AC, Downie LE. Preliminary Validation of a Food Frequency Questionnaire to Assess Long-Chain Omega-3 Fatty Acid Intake in Eye Care Practice. Nutrients. 2019;11:817. 10.3390/nu11040817 PMC6521311 30978959

[29] Labbé A, Alalwani H, Van Went C, Brasnu E, Georgescu D, Baudouin C (2012). The Relationship between Subbasal Nerve Morphology and Corneal Sensation in Ocular Surface Disease. Invest Ophthalmol Vis Sci.

[30] *R: A Language and Environment for Statistical Computing* [computer program]. Vienna, Austria: Vienna, Austria: R Foundation for Statistical Computing; 2019.

[31] Beal SL (2001). Ways to Fit a PK Model with Some Data Below the Quantification Limit. J Pharmacokinet Pharmacodyn.

[32] Wolffsohn JS, Arita R, Chalmers R, Djalilian A, Dogru M, Dumbleton K (2017). TFOS DEWS II Diagnostic Methodology report. Ocul Surf.

[33] Motulsky HJ, Brown RE (2006). Detecting outliers when fitting data with nonlinear regression – a new method based on robust nonlinear regression and the false discovery rate. BMC Bioinforma.

[34] Alcalde I, Íñigo-Portugués A, González-González O, Almaraz L, Artime E, Morenilla-Palao C (2018). Morphological and functional changes in TRPM8-expressing corneal cold thermoreceptor neurons during aging and their impact on tearing in mice. J Comp Neurol.

[35] Bazan NG (2006). Cell survival matters: Docosahexaenoic acid signaling, neuroprotection and photoreceptors. Trends Neurosci.

[36] Hong S, Tian H, Lu Y, Laborde JM, Muhale FA, Wang Q (2014). Neuroprotectin/protectin D1: Endogenous biosynthesis and actions on diabetic macrophages in promoting wound healing and innervation impaired by diabetes. Am J Physiol Cell Physiol.

[37] Cortina MS, He J, Russ T, Bazan NG, Bazan HE (2013). Neuroprotectin D1 restores corneal nerve integrity and function after damage from experimental surgery. Invest Ophthalmol Vis Sci.

[38] Heng LJ, Qi R, Yang RH, Xu GZ (2015). Docosahexaenoic acid inhibits mechanical allodynia and thermal hyperalgesia in diabetic rats by decreasing the excitability of DRG neurons. Exp Neurol.

[39] Silva RV, Oliveira JT, Santos BLR, Dias FC, Martinez AMB, Lima CKF (2017). Long-Chain Omega-3 Fatty Acids Supplementation Accelerates Nerve Regeneration and Prevents Neuropathic Pain Behavior in Mice. Front Pharm.

[40] Stark KD, Van Elswyk ME, Higgins MR, Weatherford CA, Salem N (2016). Global survey of the omega-3 fatty acids, docosahexaenoic acid and eicosapentaenoic acid in the blood stream of healthy adults. Prog Lipid Res.

[41] von Schacky C. Omega-3 Fatty Acids in Pregnancy—The Case for a Target Omega-3 Index. Nutrients. 2020;12:898. 10.3390/nu12040898 PMC7230742 32224878

[42] Meyer BJ (2016). Australians are not Meeting the Recommended Intakes for Omega-3 Long Chain Polyunsaturated Fatty Acids: Results of an Analysis from the 2011-2 National Nutrition and Physical Activity Survey. Nutrients.

[43] Katan MB, Deslypere JP, van Birgelen AP, Penders M, Zegwaard M (1997). Kinetics of the incorporation of dietary fatty acids into serum cholesteryl esters, erythrocyte membranes, and adipose tissue: an 18-month controlled study. J Lipid Res.

[44] Lewis EJH, Lovblom LE, Cisbani G, Chen DK, Bazinet RP, Wolever TMS (2021). Baseline omega-3 level is associated with nerve regeneration following 12-months of omega-3 nutrition therapy in patients with type 1 diabetes. J Diabetes Complications.

[45] Belmonte C, Acosta MC, Gallar J (2004). Neural basis of sensation in intact and injured corneas. Exp Eye Res.

[46] Patel DV, Tavakoli M, Craig JP, Efron N, McGhee CN (2009). Corneal sensitivity and slit scanning in vivo confocal microscopy of the subbasal nerve plexus of the normal central and peripheral human cornea. Cornea.

[47] Misra SL, Craig JP, Patel DV, McGhee CN, Pradhan M, Ellyett K (2015). In vivo confocal microscopy of corneal nerves: An ocular biomarker for peripheral and cardiac autonomic neuropathy in type 1 diabetes mellitus. Invest Ophthalmol Vis Sci.

[48] Breiner A, Lovblom LE, Perkins BA, Bril V (2014). Does the prevailing hypothesis that small-fiber dysfunction precedes large-fiber dysfunction apply to type 1 diabetic patients?. Diab Care.

[49] Murphy PJ, Patel S, Kong N, Ryder REJ, Marshall J (2004). Noninvasive Assessment of Corneal Sensitivity in Young and Elderly Diabetic and Nondiabetic Subjects. Invest Ophthalmol Vis Sci.

[50] Pritchard N, Edwards K, Vagenas D, Russell AW, Malik RA, Efron N (2012). Corneal sensitivity is related to established measures of diabetic peripheral neuropathy. Clin Exp Optom.

[51] Belmonte C, Nichols JJ, Cox SM, Brock JA, Begley CG, Bereiter DA (2017). TFOS DEWS II pain and sensation report. Ocul Surf.

[52] Alamri AS, Brock JA, Herath CB, Rajapaksha IG, Angus PW, Ivanusic JJ (2019). The Effects of Diabetes and High-Fat Diet on Polymodal Nociceptor and Cold Thermoreceptor Nerve Terminal Endings in the Corneal Epithelium. Invest Ophthalmol Vis Sci.

[53] Quattrini C, Tavakoli M, Jeziorska M, Kallinikos P, Tesfaye S, Finnigan J (2007). Surrogate markers of small fiber damage in human diabetic neuropathy. Diabetes.

