# Supplementary material for: Association between systemic omega-3 polyunsaturated fatty acid levels, and corneal nerve structure and function
Source: Eye (Lond). 2022 Sep 26;37(9):1866–73. doi: 10.1038/s41433-022-02259-0 (PMC10275886; doi:10.1038/s41433-022-02259-0)
Supplement: Supplementary file 1 — Supplemental Tables and Figure [file 41433_2022_2259_MOESM1_ESM.pdf]

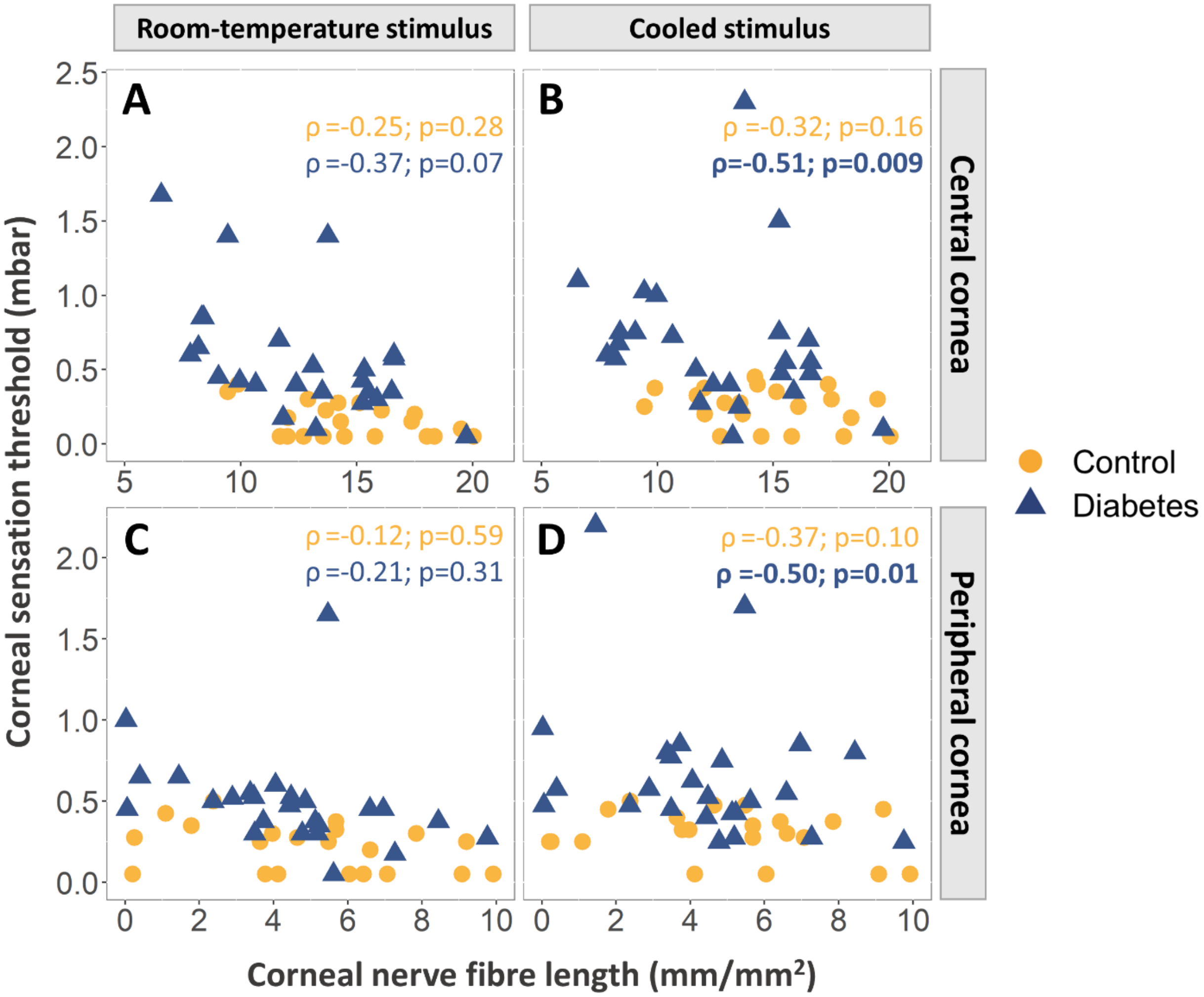

Supplemental Figure S1. Comparison of corneal structure-function relationships in the (A-B) central and (C-D) peripheral cornea in people with diabetes and healthy controls. Spearman's correlation coefficient (ρ) is reported for the relationship between corneal nerve fibre length (CNFL) and corneal sensation thresholds to room-temperature and cooled stimuli.

Supplemental Table S1. Multiple linear regression models for variables predicting central corneal sensation thresholds to room-temperature air stimuli (log transformed)

Variable	B (95% CI)	β	p-value
**Model 1: Omega-3 Index**			
Omega-3 index, %	-0.01 (-0.10 to 0.08)	-0.03	0.83
**Age, years**	**0.01 (0.001 to 0.01)**	**0.31**	**0.025**
**Diabetes, present**	**0.52 (0.30 to 0.75)**	**0.61**	**<0.001**
R=0.66. R^2=0.44. F=4.37. p=0.001.			
**Model 2: Systemic EPA levels**			
EPA levels, %	0.002 (-0.17 to 0.17)	0.003	0.98
**Age, years**	**0.01 (0.001 to 0.01)**	**0.30**	**0.03**
**Diabetes, present**	**0.53 (0.30 to 0.75)**	**0.61**	**<0.001**
R=0.66. R^2=0.44. F=4.36. p=0.001.			
**Model 3: Systemic DHA levels**			
DHA levels, %	-0.03 (-0.25 to 0.19)	-0.04	0.79
**Age, years**	**0.01 (0.001 to 0.01)**	**0.30**	**0.03**
**Diabetes, present**	**0.52 (0.30 to 0.75)**	**0.60**	**<0.001**
R=0.66. R^2=0.44. F=4.38. p=0.001.			
**Model 4: Total omega-6 fatty acid levels**			
Total omega-6 levels, %	-0.01 (-0.03 to 0.02)	-0.08	0.59
**Age, years**	**0.01 (<0.001 to 0.01)**	**0.28**	**0.04**
**Diabetes, present**	**0.49 (0.24 to 0.75)**	**0.57**	**<0.001**
R=0.67. R^2=0.44. F=4.43. p=0.001.			

Additional variables in the model not found to be related to CNFL are sex, presence of dry eye diseases, tear osmolarity (mOsmol/L) and Norfolk Quality of Life-Diabetic Neuropathy questionnaire score. **B,** unstandardised regression coefficient. **β,** standardised regression coefficient. **Abbreviations**: CI, confidence interval; DHA, docosahexaenoic acid; EPA, eicosapentaenoic acid; Norfolk QoL-DN, Norfolk Quality of Life-Diabetic Neuropathy questionnaire; PUFAs, polyunsaturated fatty acids.

Supplemental Table S2. Multiple linear regression models for variables predicting central corneal sensation thresholds to cooled air stimuli (log transformed)

Variable	B (95% CI)	β	p-value
**Model 1: Omega-3 Index**			
Omega-3 index, %	-0.05 (-0.14 to 0.05)	-0.11	0.36
Age, years	0.004 (-0.003 to 0.01)	0.14	0.26
**Diabetes, present**	**0.64 (0.41 to 0.86)**	**0.67**	**<0.001**
R=0.72. R^2=0.52. F=6.07. p<0.001.			
**Model 2: Systemic EPA levels**			
EPA levels, %	-0.05 (-0.23 to 0.12)	-0.07	0.55
Age, years	0.004 (-0.003 to 0.01)	0.15	0.24
**Diabetes, present**	**0.64 (0.41 to 0.87)**	**0.68**	**<0.001**
R=0.72. R^2=0.52. F=5.92. p<0.001.			
**Model 3: Systemic DHA levels**			
DHA levels, %	-0.09 (-0.31 to 0.14)	-0.10	0.44
Age, years	0.003 (-0.003 to 0.01)	0.13	0.29
**Diabetes, present**	**0.63 (0.40 to 0.86)**	**0.66**	**<0.001**
R=0.72. R^2=0.52. F=5.99. p<0.001.			
**Model 4: Total omega-6 fatty acid levels**			
Total omega-6 levels, %	0.002 (-0.02 to 0.02)	0.02	0.88
Age, years	0.004 (-0.003 to 0.01)	0.14	0.29
**Diabetes, present**	**0.65 (0.39 to 0.91)**	**0.68**	**<0.001**
R=0.72. R^2=0.51. F=5.83. p<0.001.			

Additional variables in the model found not to be related to CNFL are sex, presence of dry eye diseases, tear osmolarity (mOsmol/L) and Norfolk Quality of Life-Diabetic Neuropathy questionnaire score. **B,** unstandardised regression coefficient. **β,** standardised regression coefficient. **Abbreviations**: CI, confidence interval; DHA, docosahexaenoic acid; EPA, eicosapentaenoic acid; Norfolk QoL-DN, Norfolk Quality of Life-Diabetic Neuropathy questionnaire; PUFAs, polyunsaturated fatty acids.

Supplemental Table S3. Multiple linear regression models for variables predicting total dendritic cell density (log transformed)

Variable	B (95% CI)	β	p-value
**Model 1: Omega-3 Index**			
Omega-3 index, %	0.02 (-0.11 to 0.14)	0.05	0.79
Age, years	0.003 (-0.006 to 0.011)	0.10	0.51
Diabetes, present	**0.08 (-0.25 to 0.41)**	**0.08**	**0.62**
R=0.24. R^2=0.06. F=0.42. p=0.87.			
**Model 2: Systemic EPA levels**			
EPA levels, %	0.10 (-0.15 to 0.34)	0.13	0.43
Age, years	0.002 (-0.007 to 0.011)	0.08	0.62
Diabetes, present	0.073 (-0.25 to 0.40)	0.07	0.65
R=0.27. R^2=0.07. F=0.51. p=0.80.			
**Model 3: Systemic DHA levels**			
DHA levels, %	-0.07 (-0.38 to 0.24)	-0.08	0.65
Age, years	0.003 (-0.006 to 0.011)	0.10	0.52
Diabetes, present	0.067 (-0.261 to 0.396)	0.07	0.68
R=0.25. R^2=0.06. F=0.44. p=0.85.			
**Model 4: Total omega-6 fatty acid levels**			
Total omega-6 levels, %	0.02 (-0.1 to 0.05)	0.23	0.22
Age, years	0.004 (-0.004 to 0.01)	0.16	0.33
Diabetes, present	0.18 (-0.18 to 0.54)	0.19	0.31
R=0.30. R^2=0.09. F=0.68. p=0.67.			

Additional variables in the model found not to be related to CNFL are sex, presence of dry eye diseases, and Norfolk Quality of Life-Diabetic Neuropathy questionnaire score. **B,** unstandardised regression coefficient. **β,** standardised regression coefficient. **Abbreviations**: CI, confidence interval; DHA, docosahexaenoic acid; EPA, eicosapentaenoic acid; Norfolk QoL-DN, Norfolk Quality of Life-Diabetic Neuropathy questionnaire; PUFAs, polyunsaturated fatty acids.
